# Risk Factors for Failure of Hard Palate Mucoperiosteal Flap Repair of Acquired Oronasal Communication in Dogs: A Pilot Study

**DOI:** 10.3389/fvets.2021.762842

**Published:** 2021-11-03

**Authors:** Kendall Taney, Mark M. Smith, Nathan P. Cummings, Alicia J. Lozano

**Affiliations:** ^1^Center for Veterinary Dentistry and Oral Surgery, Gaithersburg, MD, United States; ^2^Maxtena, Inc., Rockville, MD, United States; ^3^Center for Biostatistics and Health Data Science, Department of Statistics, Virginia Tech, Roanoke, VA, United States

**Keywords:** palate, oral, maxillofacial, flap, surgery, oncology, dog, fistula

## Abstract

The objective of this retrospective pilot study was to describe potential risk factors for failure of hard palate mucoperiosteal flaps (HPF) transposed for closure of oronasal communication. Dogs (*n* = 28) with acquired oronasal communication defects were included in the study population. Functional success of an HPF was determined by visual inspection at the last examination and lack of clinical signs. Risk factors for HPF failure including age, sex, body weight, presence of neoplasia at the time of surgery, presence of neoplasia after surgery due to incomplete or narrow margins, use of CO_2_ laser, previous surgeries in the same location, HPF blood supply, size of the HPF as a percentage of the total area of the hard palate mucoperiosteum, and distance traveled by the apex of the HPF were evaluated using descriptive statistics and unadjusted logistic regression modeling. Seven out of 28 (25%) hard palate flap procedures resulted in persistent oronasal communication and were considered failures. Body weight (Median: 17 vs. 25 kg, OR = 0.94, 80% CI = 0.90, 0.99), presence of neoplasia at the time of surgery (86 vs. 57%, OR = 4.50, 80% CI = 1.01, 20.06), HPF area (Median: 0.49 vs. 0.41, OR = 84.40, 80% CI = 1.66, 4,298) and apex travel distance (Median: 2.06 vs. 0.67, OR = 5.15, 80% CI = 2.14, 12.38) were associated with flap failure. Within this sample, the presence of neoplasia at the time of initial surgery, increasing the area of the HPF, and distance traveled by the HPF apex were associated with a greater odds of HPF failure. Further studies with larger sample sizes are needed to confirm repeatability of these results. HPFs remain a viable surgical option for closure of oronasal communication. Careful surgical planning, strict adherence to surgical principles, and awareness of anatomical limitations can increase the likelihood of success.

## Introduction

Defects in the maxillary, incisive, and palatine bones that result in oronasal communication can develop from numerous conditions and are characterized as congenital or acquired in origin (1–7). Acquired defects may result from treatment of neoplasia, periodontal disease, erosive disorders of the oral cavity, previous surgery or radiation therapy, or traumatic injuries (1–7). Malocclusion, especially where the mandibular canine teeth chronically contact the hard palate can also result in oronasal communication (4). Surgical repair of oronasal communication is generally indicated to allow for normal function and improved quality of life. Without repair patients may experience chronic upper respiratory infections, sinusitis, food impaction, failure to thrive, and aspiration pneumonia (1–7). Primary surgical repair of oral cavity defects presents challenges due to the paucity of redundant tissue. The anatomical nature of the region limits the ability of primary closure from immediately adjacent tissues surrounding the defect. Hard palate mucoperiosteal flaps (HPF) can provide robust tissue for primary surgical repair of acquired oronasal communication (1–7).

The incisive bones, palatine processes of the maxillary and palatine bones and the associated soft tissue covering make up the structure collectively known as the hard palate (4). The soft tissue blood supply of the hard palate is delivered by the right and left major palatine arteries. The arteries pass through the major palatine canals and the major palatine foramina to enter the soft tissue on the dorsal surface at approximately the level of the maxillary fourth premolar teeth bilaterally. In the sagittal plane the major palatine foramen is approximately halfway between the lingual border of the maxillary fourth premolar tooth and midline bilaterally. Individual patients can have anatomical variations so the area should be approached with caution to avoid accidental transection of the major palatine artery (4, 8). As the major palatine artery continues rostrally, it gives off many small branches and anastomoses with both the lateral nasal artery and the contralateral major palatine artery (8).

The HPF blood supply comes directly from the major palatine artery and smaller random vessels (1–8). Basic surgical principles including knowledge of anatomy, tension-free closure, preserving adequate blood supply, and delicate tissue handling are essential for successful healing (1–7). An HPF can be carefully developed as an island for greater mobility and rotated up to 180° without compromising blood supply (9–11). Dehiscence is the most common complication of oronasal fistula repair (4, 6, 7). Previous studies have listed various reasons for mucosal flap dehiscence such as excessive tension, insufficient bony support underlying the suture line, infection, compromised blood supply, inadequate tissue reservoir for flap development, poor flap design, and poor surgical execution (3, 4, 6, 7). However, the axial nature of an HPF with direct blood supply from the major palatine artery makes it useful for maxillofacial reconstruction.

The objective of this retrospective pilot study was to identify potential risk factors for persistence of oronasal communication in cases where HPFs were used for closure. Multiple risk factors were considered as having potential for HPF failure including age, sex, body weight, presence of neoplasia at the surgical site at the time of initial surgery confirmed by histopathology, presence of neoplasia after surgery as indicated by an incomplete or narrow margin, CO_2_ laser usage for mucosal incisions, previous surgery at the same location, HPF blood supply, HPF area as a percentage of the total hard palate mucoperiosteum area, and distance traveled by the HPF apex. Based on previous studies and oral and maxillofacial surgical principles, our concerns were that neoplasia presence, CO_2_ laser usage, previous surgery, absence of direct arterial blood supply to the HPF, or greater HPF apex travel distance could contribute to HPF failure (2, 11, 12).

## Materials and Methods

Medical records (2006-2020) at the Center for Veterinary Dentistry and Oral Surgery were searched for patients with acquired oronasal communication defects repaired using HPF transposition. Patients were excluded if there was no documented follow-up examination or a clear photographic view of the final surgical closure, maxillary canine teeth, and total area of the hard palate. Of 33 cases considered, five did not meet the inclusion criteria. Two cases were lost completely to follow-up after surgery. Two cases had rapid tumor recurrence and were euthanized within 2 weeks of surgery. One case did not have clear surgical photographs for measurement but was examined at our facility 2 weeks postoperatively with excellent healing of the surgical site noted in the medical record. Twenty-eight cases met the inclusion criteria.

**Figure 1:**
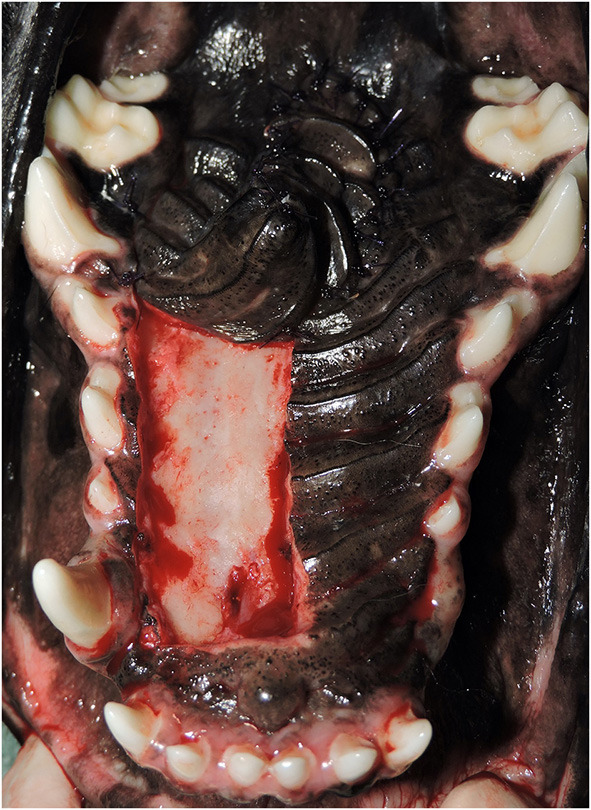
Example photograph of final closure used for all measurements. A single, clear photograph from each case showing the entire hard palate and hard palate mucoperiosteal flap (HPF) is used to make all computerized measurements for estimation of the total hard palate area, flap size, and distance traveled by the HPF.

Most of the data needed to investigate each risk factor was obtained from the medical record. The retrospective study design presented challenges when exact measurements of HPF areas and flap apex travel distances were not noted in the record. To overcome this limitation, photographs of the hard palate with final HPF placement were used to make computerized measurements. Variations in camera focal distance, optical zoom setting, and angle prevented absolute measurements from the photographs. Additionally, differences in patient size would make absolute measurements between cases difficult to compare. A computerized image analyzer developed at the National Institutes of Health (ImageJ, version 1.51) was used for feature size normalization and feature measurement. All measurements were performed by one investigator (KT). A single intraoperative photograph was selected that provided a clear view of the hard palate, maxillary canine teeth, and final location of the HPF apex. The same photograph was used for all measurements (Figure 1).

**Figure 2:**
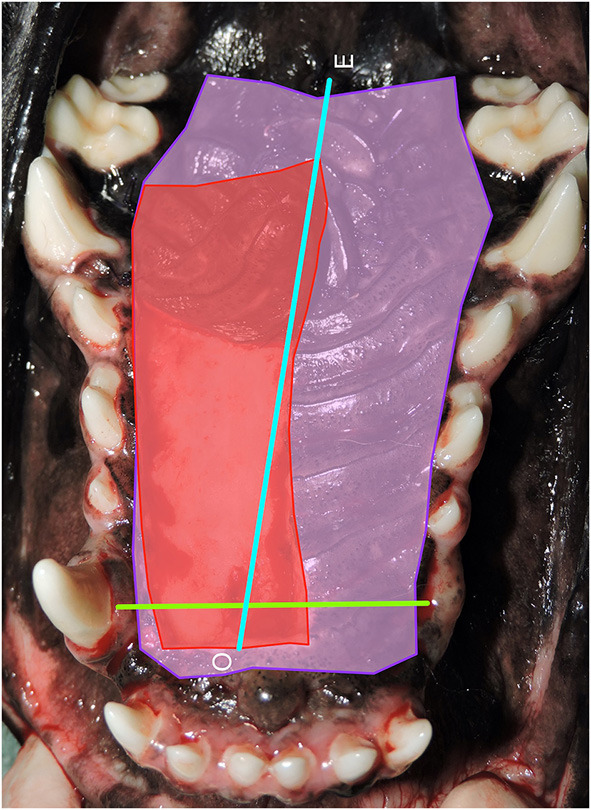
Step 1: Hard palate measurements. Photograph of the area of the hard palate with all measurements used for analysis. Purple shaded area: estimated total hard palate mucoperiosteal flap (HPF) area (pixels^2^) utilized in the repair. Red shaded area: standard unit for each patient that could be used to evaluate the significance of the distance traveled by the apex of the hard palate mucoperiosteal flap. Green line: The distance between the base of the canine teeth on the palatal surface was measured in pixels for each patient and used as normalized palatal length factor (Lp) in order to compare across species, breed, and relative patient size. Blue line: Determination of the relative distance traveled by the hard palate mucoperiosteal flap (HPF) by measuring from the original location of the apex of the HPF to the final location resulting in another pixel value. O, origin of flap apex; E, endpoint of flap apex.

The measurement and evaluation procedures were divided into six steps (Figure 2). The first step estimated the total hard palate area. This area was estimated by drawing lines around the anatomical landmarks delineating the hard palate mucoperiosteum. Hard palate mucoperiosteum anatomical landmarks used for measurement were the rostral border of the rugae just caudal to the incisive papilla, the junction of the rugae and palatal attached gingiva laterally, and the caudal border of the rugae at the level of the maxillary second molar teeth. This area represented the typical tissue available for HPF development and all measurements were calculated by the software in pixels (Purple shaded area). The second step estimated the total hard palate flap area utilized in the repair by drawing lines around the borders of the hard palate flap at its origin (Red shaded area). This value was also calculated in pixels. The third step calculated the hard palate flap area ratio by dividing the HPF area in pixels by the total hard palate flap area in pixels. Step four determined a standard unit of length for normalization of the distance traveled by the HPF apex. The distance between the base of the canine teeth on the palatal surface was selected as the normalization factor and was calculated in pixels. This normalized palatal length factor (Lp) allowed comparison across species, breed, and relative patient size (Green line). If a canine tooth was missing the investigator measured to the expected anatomical location of the tooth. The fifth step determined the relative distance traveled by the HPF by measuring from the original location of the HPF apex to its final location resulting in another pixel value (Blue line). Step six provided the final measurement of the HPF travel distance ratio by dividing the HPF travel distance by the palatal length factor.

**Figure 3:**
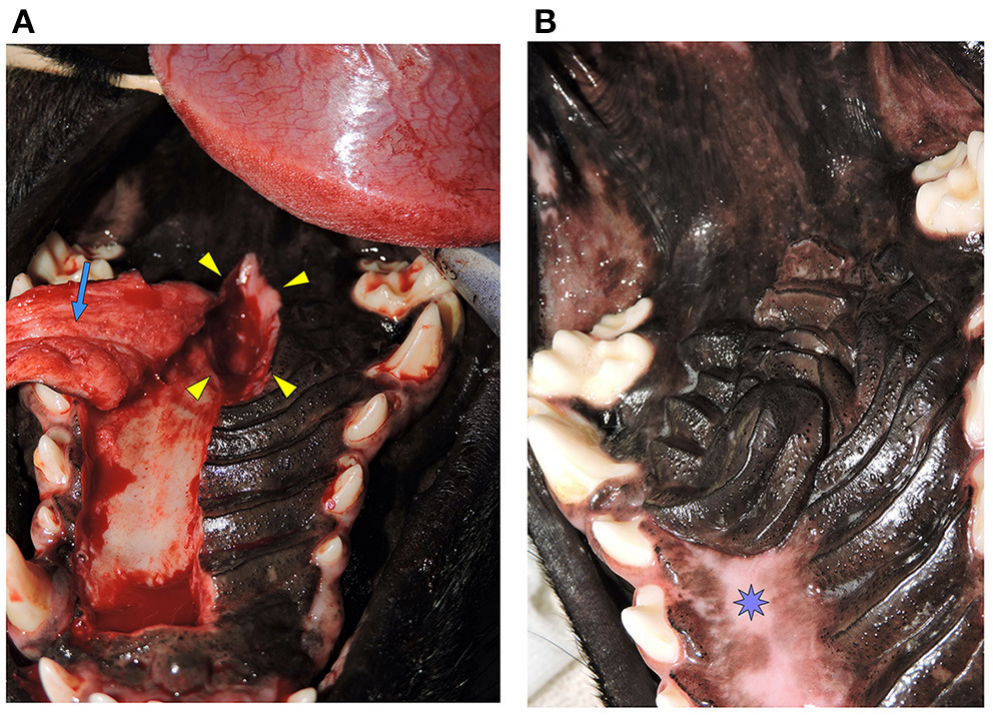
**(A)** Oronasal communication. Intraoperative photograph showing the full-thickness defect in the hard palate (Arrowheads). A hard palate mucoperiosteal flap has been elevated in preparation for closure (Arrow). This is the same patient from Figure 1. **(B)** Documentation of healing. Photograph showing successful healing of the hard palate mucoperiosteal flap repair. The denuded hard palate bone has re-epithelialized (Star). This is the same patient from Figure 1.

**Figure 4:**
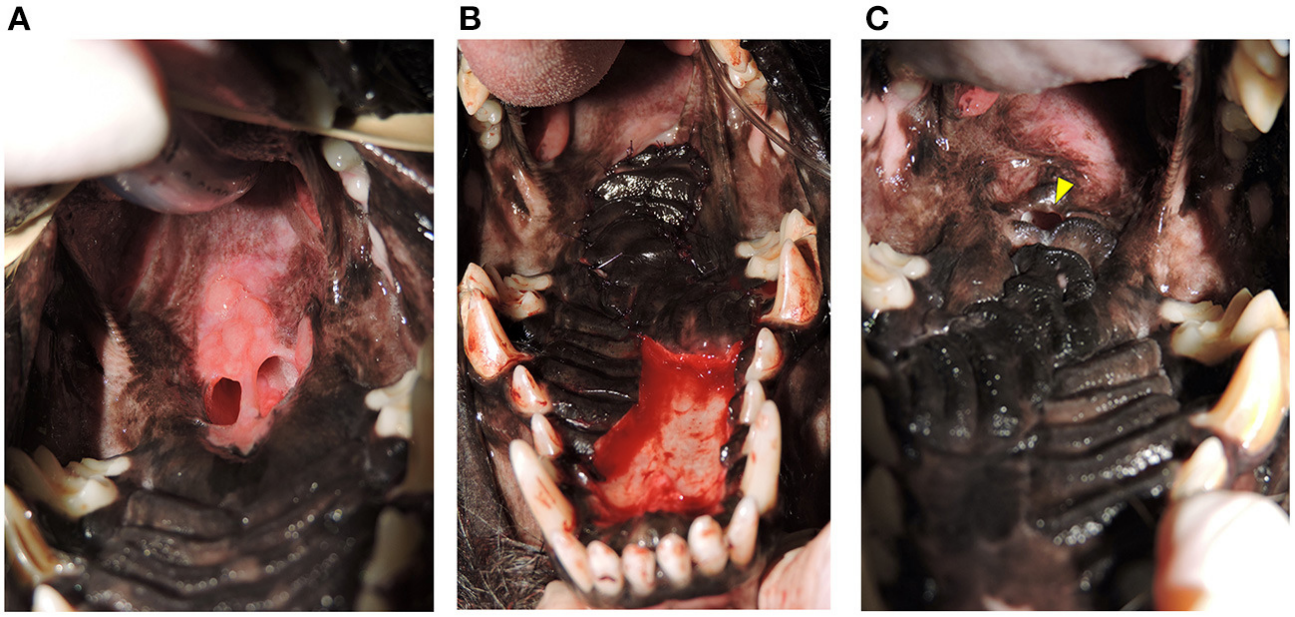
**(A)** Oronasal communication from eosinophilic granuloma complex. Photograph showing an oronasal communication near the junction of the hard and soft palate prior to repair. **(B)** Island Mucoperiosteal Flap Repair. Photograph showing the same patient from **(A)** after closure with an island design hard palate mucoperiosteal flap. **(C)** Failure of flap repair. Photograph showing the same patient from **(A,B)**. A persistent oronasal communication remains indicating partial failure of the island hard palate mucoperiosteal flap (arrowhead).

Functional success was determined by visual inspection at the most recent examination and lack of reported symptoms such as sneezing or nasal discharge. Unlike skin flaps that are readily visualized by the clinician or owner during the peri-postoperative period whereby devitalization is readily apparent, flaps in the oral cavity are more difficult to evaluate in circumstances of procedure failure. Owners were cautioned against opening the mouth to avoid any untoward tension on the flap during the early phases of wound healing. No oral examination was performed until the scheduled 2-week postoperative examination unless the patient had clinical signs of oronasal communication recurrence. Photographs were obtained of the healed surgical site at a minimum of 14 days postoperatively in all but one case. This patient was examined at our facility at 2 and 42 weeks postoperatively and documented to have complete healing of the surgical site. Additionally, in an email communication approximately 78 weeks postoperatively, the owner stated that the patient was continuing to do well with no clinical signs of oronasal communication. If there was documentation of mucosal incisional healing and no clinical signs at 14 days this was considered the minimum follow-up for inclusion in the study group based on previous studies showing adequate oral wound tensile strength at 14 days (13–16) [Figures 3, 4—before and after a successful case (Figures 3A,B) and a failure case (Figures 4A–C)]. Repeat examinations by the surgeon were performed at longer intervals in as many patients as possible. For those patients that were deceased at the time of this study medical records from the primary veterinarian or other specialists were obtained to determine if there was physical or clinical evidence of HPF failure at any time.

Descriptive statistics in the overall sample and by HPF failure status (yes vs. no) were used to characterize proposed risk factors including: age (years), sex (male vs. female), body weight (kilograms), postoperative follow-up period in the overall sample and by HPF failure status (yes vs. no), presence of neoplasia at the time of surgery (yes vs. no) or after surgery based on surgical margins (≤5 mm or was incomplete vs. >5 mm), use of CO_2_ laser (yes vs. no), previous surgeries (yes vs. no), blood supply to the HPF (at least one greater palatine artery providing blood supply vs. none), HPF size as a percentage of the total hard palate mucoperiosteum area, distance traveled by the HPF apex (Lp units). Continuous variables were described using means, standard deviations, medians, interquartile ranges, and ranges. Normality of continuous variables was assessed quantitively using Shapiro-Wilk tests and visually using histograms. Categorical variables were described using frequencies and percentages. Unadjusted logistic regression models were used to examine the individual impact of each proposed risk factor on the odds of HPF failure. Odds ratios and their 80% confidence intervals were generated as effect sizes. Note that 80% confidence intervals were used due to the pilot nature of the study (17). Odds ratios with 80% confidence intervals that do not include 1 indicated statistical significance at the 0.20 level. As a pilot study, we recognize the small sample size (*n* = 28) and that the purpose of this study is to estimate effect sizes (i.e., odds ratios) to inform future studies (18). All analyses were completed using SAS version 9.4 (SAS Institute Inc., Cary, NC).

## Results

Table 1 summarizes the proposed risk factors by HPF failure status. The mean age was 8.3 years (SD = 3.6). Of 28 total dogs, 14 were male and 14 were female and all dogs except for one male dog were neutered. Seven out of 28 HPF procedures failed (25%), all of which occurred in neutered dogs (four males and three females). Median body weight across the entire study group was 23.2 kg (range, 4.3-47.7 kg), with a mean weight of 22.9 kg (SD = 12.9). For every kilogram increase in body weight, the odds of HPF failure decreased by 6% (median: 17 vs. 25 kg, OR = 0.94, 80% CI = 0.90, 0.99). No significant differences in age [median: 10 vs. 9, Odds Ratio (OR) = 1.13, 80% CI = 0.95, 1.34] or sex (43 vs. 52% female, OR = 1.47, 80% CI = 0.47, 4.53) were found among cases with HPF failure vs. none in all animals (Table 1).

**Table 1:** Sample characteristics by HPF failure status.

	**All dogs (*n* = 28)**	**HPF repair failure (*n* = 7)**	**No HPF failure (*n* = 21)**	**Unadjusted odds ratio (80% CI)**
**Age (Years)**				1.13 (0.95, 1.34)
Mean (SD)	8.29 (3.59)	9.36 (3.12)	7.94 (3.73)	
Median (Q1, Q3)	9.00 (5.50, 11.00)	10.00 (6.00, 12.00)	9.00 (5.00, 10.00)	
Min, Max	0.75, 14.00	4.50, 13.00	0.75, 14.00	
**Sex**, ***n*** **(%)**				
Female	14 (50%)	3 (43%)	11 (52%)	-Reference-
Male	14 (50%)	4 (57%)	10 (48%)	1.47 (0.47, 4.53)
**Body weight (Kg)**				0.94 (0.90, 0.99)
Mean (SD)	22.91 (12.86)	16.79 (8.74)	24.94 (13.52)	
Median (Q1, Q3)	23.15 (11.03, 35.60)	17.20 (7.17, 23.70)	25.45 (13.60, 37.90)	
Min, Max	4.27, 47.70	6.50, 29.00	4.27, 47.70	
**Postoperative follow-up period (Weeks)**				0.99 (0.98, 1.01)
Mean (SD)	47.18 (58.67)	36.14 (39.17)	50.86 (64.26)	
Median (Q1, Q3)	16.00 (9.50, 59.00)	16.00 (9.00, 54.00)	16.00 (10.00, 64.00)	
Min, Max	2.00, 236.00	8.00, 117.00	2.00, 236.00	
**Presence of neoplasia at initial surgery**, ***n*** **(%)**				
Yes	18 (64%)	6 (86%)	12 (57%)	4.50 (1.01, 20.06)
No	10 (36%)	1 (14%)	9 (43%)	-Reference-
**Surgical margins >5 mm**, ***n*** **(%)**				
Yes	15 (54%)	3 (43%)	12 (57%)	0.56 (0.18, 1.74)
No	13 (46%)	4 (57%)	9 (43%)	-Reference-
**Use of CO**_**2**_ **Laser**, ***n*** **(%)**				
Yes	19 (68%)	6 (86%)	13 (62%)	3.69 (0.82, 16.53)
No	9 (32%)	1 (14%)	8 (38%)	-Reference-
**Previous surgeries**, ***n*** **(%)**				
Yes	7 (25%)	0	7 (33%)	0.13 (0.02, 1.04)
No	21 (75%)	7 (100%)	14 (67%)	-Reference-
**At least one greater palatine artery providing blood supply**, ***n*** **(%)**				
Yes	25 (89%)	7 (100%)	18 (86%)	2.84 (0.28, 28.54)
No	3 (11%)	0	3 (14%)	-Reference-
**Size of HPF as percentage of total area of hard palate mucoperiosteum**				84.40 (1.66, 4298)
Mean (SD)	0.45 (0.18)	0.54 (0.08)	0.43 (0.19)	
Median (Q1, Q3)	0.48 (0.37, 0.57)	0.49 (0.48, 0.64)	0.41 (0.32, 0.54)	
Min, Max	0.10, 0.83	0.47, 0.68	0.10, 0.83	
**Distance traveled by the apex of the HPF (Lp units)**				5.15 (2.14, 12.38)
Mean (SD)	1.18 (0.81)	1.89 (0.67)	0.95 (0.71)	
Median (Q1, Q3)	0.88 (0.50, 1.80)	2.06 (1.39, 2.26)	0.67 (0.46, 1.22)	
Min, Max	0.18, 2.90	0.86, 2.90	0.18, 2.60	

*SD, standard deviation; Q1, lower quartile; Q3, upper quartile; Min, minimum; Max, maximum; CI, confidence interval*.

**Figure 5:**
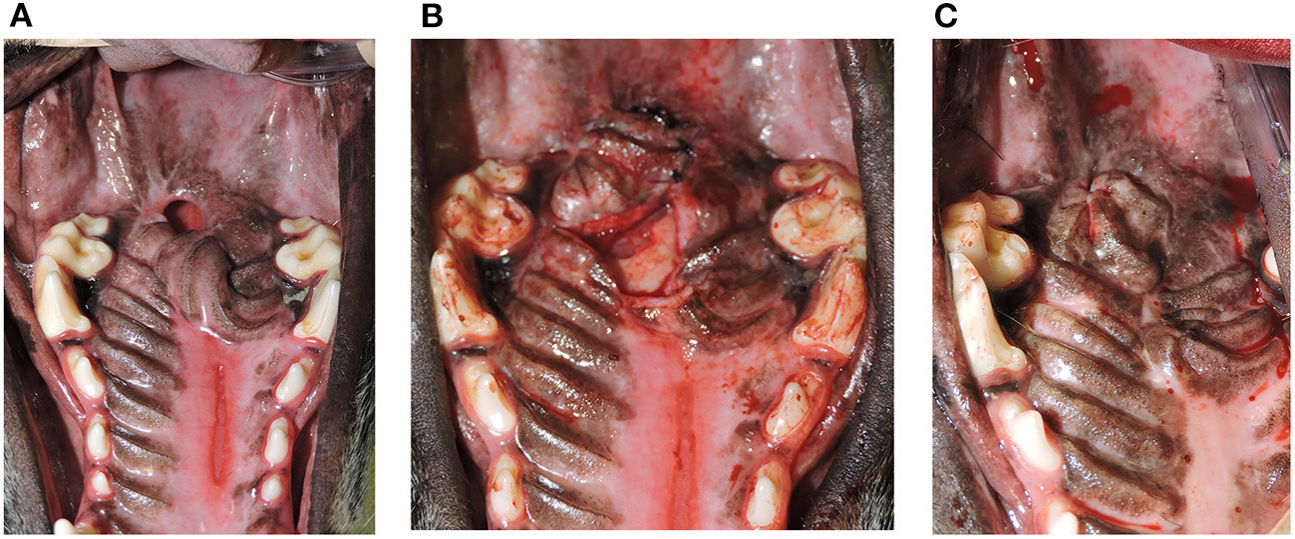
**(A)** Persistent oronasal communication. Photograph depicting a remaining defect after hard palate mucoperiosteal flap repair of a caudal oronasal communication secondary to removal of an eosinophilic granuloma. **(B)** Repair of persistent oronasal communication. Photograph showing the second procedure performed to close the oronasal communication in the patient from **(A)**. **(C)** Documentation of successful repair. Photograph showing the 1-year postoperative outcome and complete closure of the oronasal communication in the patient from **(A,B)**.

Six patients had additional surgical repair(s) of persistent oronasal communication that ultimately healed (Figures 5A–C). One patient had tumor recurrence 3 weeks postoperatively and no additional surgery was performed. The median postoperative follow-up period was 16 weeks with a range of 2-236 weeks and a mean of 47 weeks (Table 1), with no differences between those with vs. no HPF failure (Median: 16 vs. 16 weeks, OR = 0.99, 80% CI = 0.98, 1.01). In four cases the follow-up interval was shorter than preferred (2-4 weeks). One of the HPF failure cases had a follow-up period of 4 weeks. Reasons for shorter follow-up included rapid tumor recurrence (two cases), lack of response from the owner (one case), and death from an unrelated cause 1 day prior to the 8-week recheck in one case.

Among all cases, 18 (64%) had neoplasia and six of those cases had HPF failure. Three patients had malignant melanoma and one patient had a multicentric plasmacytoma. One patient with melanoma had histopathologic margins >10 mm. Histopathology in another patient with melanoma noted the dorsal margin toward the nasal cavity was 1 mm covered with a thin layer of bone and all other margins >10 mm. The remaining melanoma patient and the patient with plasmacytoma had histopathologic margins of 2 mm at the narrowest point. One patient's initial biopsy indicated squamous cell carcinoma of the left caudal maxilla, but the final histopathology returned a diagnosis of an odontogenic cyst with >10 mm margins. The patient was evaluated over 2 years after maxillectomy with HPF closure and had no indication of neoplasia recurrence. Patients with presence of neoplasia prior to surgery had a 4.5-fold odds of HPF failure compared to those without (86 vs. 57%, OR = 4.50, 80% CI = 1.01, 20.06). No significant differences in narrow (<5 mm) or incomplete margins (43 vs. 57%, OR = 0.56, 80% CI = 0.18, 1.74) were observed between cases with vs. no HPF failure among all patients (Table 1).

A CO_2_ laser was used to create surgical incisions in 19 cases (68%) and HPF failure was noted in six of those cases (86 vs. 62%, OR = 3.69, 80% CI = 0.82, 16.53). Six failures out of 19 cases represents a 31.5% failure rate. Previous surgical procedures occurred at the same location in seven cases (25%). None of those cases had an HPF failure. HPFs in this study were further described according to their blood supply. In 25 cases (89%) there was blood supply from at least one major palatine artery. Larger hard palate flaps and double split palatal u-flaps received blood supply from both major palatine arteries. In three cases, the hard palate flap received blood supply from random vessels since the major palatine artery was not incorporated in the HPF design. Five out of seven HPF failures had direct blood supply from at least one major palatine artery. No significant differences in previous surgical procedures at the same location (0 vs. 33%, OR = 0.13, 80% CI = 0.02, 1.04), and flap blood supply (100 vs. 86%, OR = 2.84, 95% CI = 0.28, 28.54) were observed between cases with HPF failure vs. not (Table 1).

Among all patients, the mean normalized flap area was 0.45 (SD = 0.18) of the estimated total hard palate area, with a median flap apex travel distance of 0.88 Lp (range, 0.2-2.9 Lp units). Differences in normalized flap area and flap apex travel distance were observed between cases with vs. no HPF failure. Specifically, normalized flap area (Median: 0.49 vs. 0.41, OR = 84.40, 80% CI = 1.66, 4,298) and flap apex travel distance (Median: 2.06 vs. 0.67, OR = 5.15, 80% CI = 2.14, 12.38) were significantly larger in cases experiencing HPF failure vs. not (Table 1).

## Discussion

The focus of this retrospective pilot study was on acquired oronasal communication that was the result of surgery, trauma, malocclusion, or inflammatory conditions. Most of the patients in this study underwent surgical resection of an oral mass that required immediate reconstruction for closure of oronasal communication.

Repair of oronasal communication can be achieved with various methods. Literature review found many described surgical techniques including primary closure with local mucosal flaps, advancement flaps, axial pattern flaps, free graft microvascular transfer, tongue flaps, auricular cartilage free grafts, artificial obturation, and HPFs (1–7, 9–11, 19–33).

**Figure 6:**
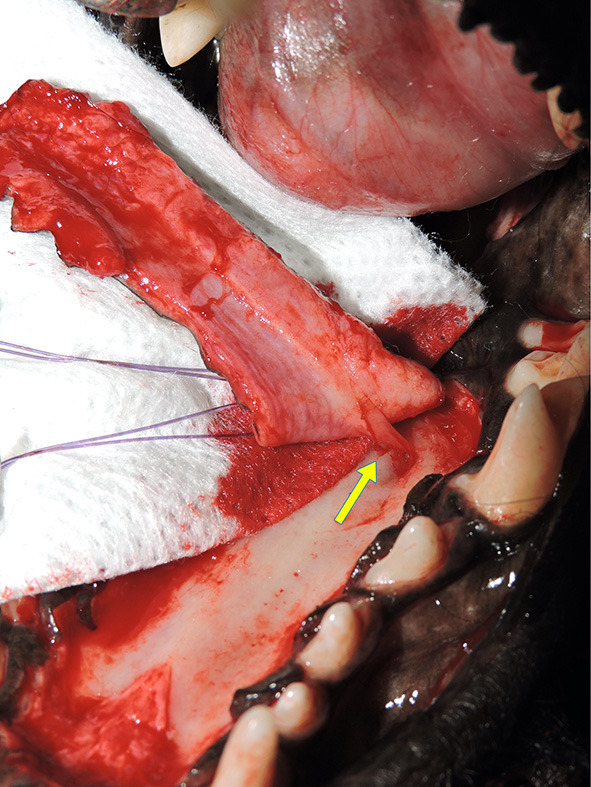
Preservation of blood supply. Photograph showing the major palatine artery as it exits the major palatine foramen at the level of the maxillary fourth premolar tooth halfway between midline and the alveolar process (arrow).

Blood supply preservation is an integral surgical principle to honor during development of an HPF (1–7). With direct blood supply from the major palatine artery, the HPF can function similarly to an axial pattern flap and provide robust tissue for immediate defect reconstruction as documented by its island application and in the one case reported here (2, 9–11) (Figure 6).

**Figure 7:**
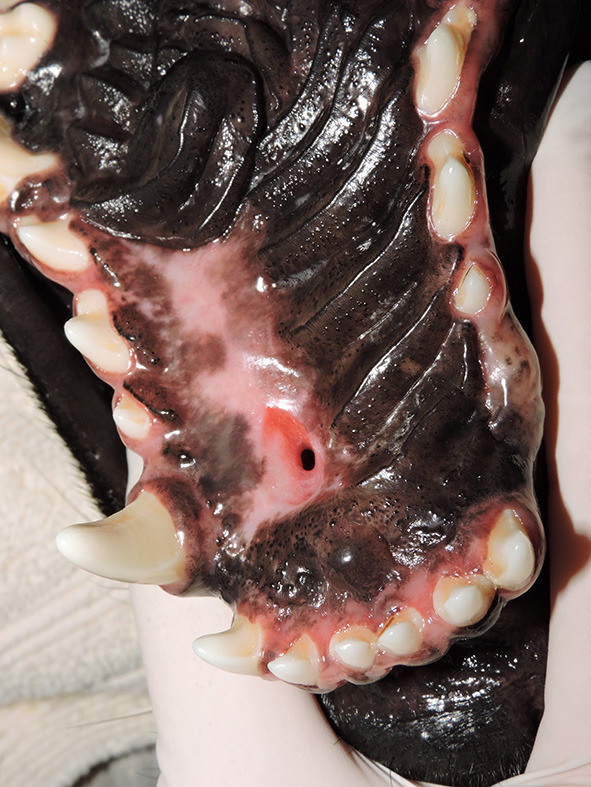
Full vs. partial-thickness flaps. Photograph showing healing following full-thickness elevation of a hard palate mucoperiosteal flap (HPF). Note the oronasal communication at the left palatine fissure as a complication of full-thickness rostral elevation of the HPF. Partial-thickness elevation of the flap in the region of the incisive papilla/palatine fissure may have prevented this complication.

HPFs utilized in these cases were most often full-thickness. Full-thickness flaps are developed with aggressive elevation off the bony surface of the hard palate using a large periosteal elevator, avoiding the major palatine artery as it exits the major palatine foramen. Partial-thickness flaps are developed utilizing a scalpel blade. If this blade technique is not performed carefully it can lead to iatrogenic damage to the flap and/or blood supply (34–36). Blood supply preservation may be best achieved by elevating a full-thickness HPF to include the connective tissue, particularly in cases where the greater palatine artery is not incorporated in the flap (2, 4, 37). An expected complication of full-thickness flap transposition is maxillary and/or palatine bone exposure that requires healing by re-epithelization. In the absence of other surgical options, this complication is tolerable and the denuded hard palate should re-epithelialize within 3 weeks (4–7, 34–36). One area which may benefit from partial-thickness HPF development is the rostral hard palate over the palatine fissures. Disruption of this area results in controllable hemorrhage and exposure of the palatine fissures which may result in oronasal communication that may not heal by spontaneous re-epithelialization (11) (Figure 7). Modifying the full-thickness HPF to partial-thickness in the area of the incisive papilla may avoid these complications. The keratinized tissue and attachment of the hard palate mucoperiosteum *via* the lamina propria to underlying bone puts constraints on the elasticity of the HPF and limits the range of movement (4, 8, 38). As mentioned previously an HPF with direct blood supply from the major palatine artery can be rotated up to 180° using the island mucoperiosteal flap surgical technique, thus greatly increasing the range of movement (9–11).

Neoplasia at the surgical site can have a negative effect on the quality of the tissue that remains for reconstruction and often presents an aberrant blood supply (39–43). Neoplasia presence was considered to have a potential negative consequence on HPF repair viability. Six out of seven failed HPFs (86%) had neoplasia in the oral cavity at the time of surgery. Twelve other cases with neoplasia healed without issue, including those with narrow or incomplete tumor resection. In two cases an HPF repair of oronasal communication was performed for palliation in patients with gross tumor. The HPFs healed without complication. The odds of HPF failure increased by 4.5 in cases with neoplasia at the time of surgery compared to those without neoplasia. The rate of HPF failure in a neoplastic environment is clinically important and supports the principle that healthy donor and recipient tissue is needed for successful repair with an HPF. Interestingly, in two cases where the HPF failed the patients had a histopathologic diagnosis of eosinophilic granuloma complex. Erosive inflammatory disorders of the oral cavity can present an added challenge for surgical repair. It is essential that the inflammation and, ideally, the underlying condition be controlled prior to surgical intervention, or wound healing may be compromised. Patients with these inflammatory disorders may need to be on life-long immunosuppressive therapies to prevent recurrence (11). Table 2 lists the numbers of patients per condition or disease in this study for reference. None of the cases in this study had undergone radiation therapy prior to surgery. Radiation can have a significant effect on the tissues and could be expected to be a negative prognostic indicator for flap success (44, 45).

**Table 2:** Conditions represented in study group.

**Condition, *n* (%)**	**All dogs (*n* = 28)**
Malignant melanoma	8 (28.6%)
Iatrogenic	5 (17.9%)
Eosinophilic granuloma complex	2 (7.1%)
Fibrosarcoma	2 (7.1%)
Squamous cell carcinoma	1 (3.6%)
Acanthomatous ameloblastoma	1 (3.6%)
Malocclusion	1 (3.6%)
Odontogenic cyst	1 (3.6%)
Osteosarcoma	1 (3.6%)
Papillary squamous cell carcinoma	1 (3.6%)
Plasmacytoma	1 (3.6%)
Reactive bone/inflammation	1 (3.6%)
Soft tissue sarcoma	1 (3.6%)
Trauma	2 (7.1%)

Scar tissue formation and loss of tissue in failed procedures may affect the success of subsequent surgeries (3, 4, 46). Seven out of 28 patients had previous surgeries and none of these were in the failure group. Neoangiogenesis which has occurred at previous surgical sites could also have influenced blood supply and could favorably affect healing.

Use of CO_2_ laser for oral and maxillofacial surgical procedures and its effect on healing has been evaluated in previous studies (47–49). Delayed healing is a concern with usage of a CO_2_ laser for creation of mucoperiosteal incisions (2–4, 6, 46, 50). CO_2_ laser was used to create surgical incisions in 19/28 cases (68%). Of those 19 cases there were six failures (31.5%) which could indicate clinical significance. Usage of CO_2_ laser was found to have a 3.69-fold increased risk of HPF failure, however the 80% CI contains 1 which would mean that this is not significant at the 0.20 level. Of the remaining 21 successful flap applications, 13 were incised with CO_2_ laser (62%). Nevertheless, the surgeon should carefully select cases and be confident in their surgical technique when using a CO_2_ laser to prevent excessive tissue damage that can negatively affect the success of the HPF procedure. Six out of seven failure cases in this study had both neoplasia at the time of surgery and usage of CO_2_ laser to create surgical incisions, thus it is not surprising that the odds ratios for these two risk factors are higher.

Determining the importance of HPF size and travel distance presented a challenge when comparing patients of multiple sizes. An HPF measuring three-centimeters in a small dog would be proportionally larger than a three-centimeter flap in a large dog with a greater area of hard palate mucoperiosteum. Similarly, an HPF traveling five centimeters in a small dog could have been moved to more anatomically caudal regions or to areas with greater motion than a flap traveling the same distance in a larger patient. To normalize variables and allow comparison between subjects, a standard measurement unit for each patient was established. The distance between the canine teeth became the palatal unit to allow for calculation of the magnitude of HPF travel distance. This transverse measurement was the most consistent measurement available for normalization from the surgical images. Calculating the percentage of the hard palate contained within the HPF provided another measure for comparison across subjects of different sizes. Ideally, exact measurements of these values at the time of surgery would have been taken and documented in the medical record. The retrospective nature of this study required the authors to develop an alternate means of measurement to evaluate the potential relationship between relative flap size, flap travel distance, and risk of failure. Use of intraoperative photographs and an image analyzer provided a repeatable method for assigning values to each feature to allow for the necessary calculations. Future studies could use exact measurements and a longitudinal normalization measurement for evaluation of HPF area and HPF apex travel distance.

Other risk factors for failure in this study were HPFs that were greater in total area and traveled the greatest distance from the donor site and these factors were found to have large odds ratios (OR = 84.40 and OR = 5.15, respectively). In some cases, HPFs were rotated up to 180°, and this factor could have caused restricted blood flow to the flap. In many cases these flaps were also transferred to an area not fully supported by bone, or to an area with higher motion than would be present at the donor site. Flaps may also have been placed over an area with greater air flow to the dorsal aspect of the flap resulting in desiccation. Single layer closure was performed in all but two cases in this study, and the cases that failed were in the former category. Previous case series studies and current literature have advocated double-layer closure where possible or staged procedures of tooth extractions followed by double-layer closure after healing of extraction sites (3, 4, 46, 50–54). A small case series evaluated outcomes of double-layer closure for hard palate defects. Cheek teeth were extracted to facilitate future development of buccal mucosal flaps. Double layer closure was performed 4-8 weeks after tooth extractions to allow for adequate tissue healing prior to closure of the defect. Three out of six cases (50%) were successful in complete closure on the first attempt (3). A similar study evaluating the outcome of surgical correction of patients with both hard and soft palate defects utilized double-layer closure in 22 out of 26 cases (85%). Partial dehiscence and formation of an oronasal fistula occurred in 13 of the 26 cases (50%), often at the junction of the hard and soft palate (46). In both studies additional surgery(ies) allowed for complete closure of the defect in almost all patients, as was the case in our study population.

In conclusion, the results of this retrospective pilot study described potential risk factors for failure of HPFs. In this pilot study, presence of neoplasia at the time of initial surgery increased the odds of HPF failure. Tissue quality and blood supply in a neoplastic environment is variable and can affect procedural success. Flaps that were larger in area, and flaps that traveled a greater distance were more likely to have been rotated, have unsupported suture lines, or were transposed to areas of higher motion, all of which may have contributed to failure. CO_2_ laser usage may have clinical significance despite having a moderate effect size in our analysis. Studies on risk factors for failure of HPFs are limited in the veterinary literature. The combination of neoplasia and CO_2_ laser usage should be further investigated, and other factors such as size and location of the defect and surgeon skill level could be added as potential risk factors in a larger study. We recognize the limitations of effect size estimates from studies with small samples, including imprecise and biased estimates (55); however, clinically meaningful effects in this sample have been described and can be used to inform larger studies that may improve the knowledge base and further the goal of reducing surgical complications and patient morbidity. Despite the complications encountered in some cases, HPFs remain a viable treatment option for oronasal communication. Careful surgical planning, strict adherence to surgical principles, and awareness of anatomical limitations can increase the likelihood of success.

## Data Availability Statement

The raw data supporting the conclusions of this article will be made available by the authors, without undue reservation.

## Ethics Statement

Ethical review and approval was not required for the animal study because retrospective study about animal patients in a clinical setting on surgical procedures consented to by animal owners. Written informed consent for participation was not obtained from the owners because no identifying information for each patient is included in this study.

## Author Contributions

KT: conception and design of the work, acquisition of data, interpretation of data, drafting and revision of the manuscript, and approval of the final version to be published. MS: study subject, revision of the manuscript, and approval of the version to be published. NC: conception and design of the work, image evaluation methodology, statistical analysis of data, interpretation of data, revision of the manuscript, and approval of the version to be published. AL: statistical analysis of data, interpretation of data, revision of manuscript, and approval of the version to be published. All authors contributed to the article and approved the submitted version.

## Funding

Research reported in this publication was supported in part by the National Center for Advancing Translational Sciences of the National Institutes of Health under Award Number UL1TR003015.

## Author Disclaimer

The content is solely the responsibility of the authors and does not necessarily represent the official views of the National Institutes of Health.

## Conflict of Interest

NC is employed by Maxtena, Inc. The remaining authors declare that the research was conducted in the absence of any commercial or financial relationships that could be construed as a potential conflict of interest.

## Publisher's Note

All claims expressed in this article are solely those of the authors and do not necessarily represent those of their affiliated organizations, or those of the publisher, the editors and the reviewers. Any product that may be evaluated in this article, or claim that may be made by its manufacturer, is not guaranteed or endorsed by the publisher.

## References

[1] Salisbury SK. Problems and complications associated with maxillectomy, mandibulectomy, and oronasal fistula repair. Probl Vet Med. (1991) 3:153-69. 1802245

[2] Sivacolundhu RK. Use of local and axial pattern flaps for reconstruction of the hard and soft palate. Clin Tech Small Anim Pract. (2007) 22:61-9. 10.1053/j.ctsap.2007.03.005 17591291

[3] Peralta S Nemec A Fiani N Verstraete FJ. Staged double-layer closure of palatal defects in 6 dogs. Vet Surg. (2015) 44:423-31. 10.1111/j.1532-950X.2014.12131.x 24476120

[4] Peralta S Manfra-Marretta S. Acquired palatal defects. In: Verstraete FJM Lommer MJ Arzi B editors. Oral and Maxillofacial Surgery in Dogs and Cats. 2nd ed. St. Louis: Elsevier (2020) p. 404-14.

[5] Smith MM. Oronasal fistula repair. Clinical Tech Sm Anim Pract. (2000) 15:243-50. 10.1053/svms.2000.21398 11270001

[6] Zacher AM Marretta SM. Oral and maxillofacial surgery in dogs and cats. Vet Clin North Am Small Anim Pract. (2013) 43:609-49. 10.1016/j.cvsm.2013.02.010 23643024

[7] Lothamer C Rawlinson JW. Palatal and oronasal defects. In: Monnet E Smeak DD editors. Gastrointestinal Surgical Techniques in Small Animals. Hoboken, NJ: Wiley Blackwell (2020). p. 71-84.

[8] Evans HE Miller ME. Miller's Anatomy of the Dog. 4th ed. St. Louis: Elsevier (2013). p. 282-4.

[9] Smith MM. Island palatal mucoperiosteal flap for repair of oronasal fistula in a dog. J Vet Dent. (2001) 18:127-9. 10.1177/089875640101800303 11968905

[10] Kim HY Hwang J Lee WJ Roh TS Lew DH Yun IS. Palatal mucoperiosteal island flaps for palate reconstruction. Arch Craniofac Surg. (2014) 15:70-4. 10.7181/acfs.2014.15.2.70 28913194 PMC5556817

[11] Woodward TM. Greater palatine island axial pattern flap for repair of oronasal fistula related to eosinophilic granuloma. J Vet Dent. (2006) 23:161-6. 10.1177/089875640602300305 17022195

[12] Kirby BM. Oral flaps. Principles, problems, and complications of flaps for reconstruction of the oral cavity. Probl Vet Med. (1990) 2:494-509. 2134609

[13] Shetty V Le AD. Oral soft tissue wound healing. In: Verstraete FJM Lommer MJ Arzi B editors. Oral and Maxillofacial Surgery in Dogs and Cats. 2nd ed. St. Louis: Elsevier (2020). p. 1-5.

[14] Hiatt WH Stallard RE Butler ED Badgett B. Repair following mucoperiosteal flap surgery with full gingival retention. J Periodontal. (1968) 39:11-6. 10.1902/jop.1968.39.1.11 5244502

[15] Wikesjö Ulf ME Selvig KA. Periodontal wound healing and regeneration. Periodontol 2000. (1999) 19:21-39. 10.1111/j.1600-0757.1999.tb00145.x 10321214

[16] Werfully S Areibi G Toner M Bergquist J Walker J Renvert S. Tensile strength, histological and immunohistochemical observations of periodontal wound healing in the dog. J Periodontal Res. (2002) 37:366-74. 10.1034/j.1600-0765.2002.01375.x 12366860

[17] Stallard N. Optimal sample sizes for phase II clinical trials and pilot studies. Stat Med. (2012) 31:1031-42. 10.1002/sim.4357. 10.1002/sim.4357 22052407

[18] Bell ML Whitehead AL Julious SA. Guidance for using pilot studies to inform the design of intervention trials with continuous outcomes. Clin Epidemiol. (2018) 10:153-7. 10.2147/CLEP.S146397 29403314 PMC5779280

[19] Marretta SM Grove TK Grillo JF. Split palatal u-flap: a new technique for repair of caudal hard palate defects. J Vet Dent. (1991) 8:5-8. 10.1177/089875649100800105 1930735

[20] Beckman BW. Split palatal u-flap for repair of caudal palatal defects. J Vet Dent. (2006) 23:267-9. 10.1177/089875640602300414 17286131

[21] Cox CL Hunt GB Cadier MM. Repair of oronasal fistulae using auricular cartilage grafts in five cats. Vet Surg. (2007) 36:164-9. 10.1111/j.1532-950X.2007.00249.x 17335424

[22] Soukup JW Snyder CJ Gengler WR. Free auricular cartilage autograft for repair of an oronasal fistula in a dog. J Vet Dent. (2009) 26:86-95. 10.1177/089875640902600203 19718972

[23] Robertson JJ Dean PW. Repair of a traumatically induced oronasal fistula in a cat with a rostral tongue flap. Vet Surg. (1987) 16:164-6. 10.1111/j.1532-950X.1987.tb00930.x 3507136

[24] Degner DA Lanz OI Walshaw R. Myoperitoneal microvascular free flaps in dogs: an anatomical study and a clinical case report. Vet Surg. (1996) 25:463-70. 10.1111/j.1532-950X.1996.tb01444.x 8923725

[25] Iller J Lanz OI Degner DA. Rectus abdominis free muscle flap for reconstruction in nine dogs. Vet Surg. (2007) 36:259-65. 10.1111/j.1532-950X.2007.00266.x 17461951

[26] Lanz OI. Free tissue transfer of the rectus abdominis myoperitoneal flap for oral reconstruction in a dog. J Vet Dent. (2001) 18:187-92. 10.1177/089875640101800402 11968900

[27] Dicks N Boston S. The use of an angularis oris axial pattern flap in a dog after resection of a multilobular osteochondroma of the hard palate. Can Vet J. (2010) 51:1274-8. 21286330 PMC2957038

[28] Nakahara N Mitchell K Straw R Kung M. Hard palate defect repair by using haired angularis oris axial pattern flaps in dogs. Vet Surg. (2020) 49:1195-202. 10.1111/vsu.13435 32452533

[29] Ishikawa Y Goris RC Nagaoka K. Use of a cortico-cancellous bone graft in the repair of a cleft palate in a dog. Vet Surg. (1994) 23:201-5. 10.1111/j.1532-950X.1994.tb00473.x 8066985

[30] Doyle CP Degner DA. Evaluation of the superior labial musculomucosal flap in dogs: an angiographic study and case report. Vet Comp Orthop Traumatol. (2019) 32:133-8. 10.1055/s-0039-1677746 30736093

[31] Edstrom EJ Smith MM. Prosthetic appliance for oronasal communication obturation in a dog. J Vet Dent. (2014) 31:108-12. 10.1177/089875641403100208 25185336

[32] Hale FA Sylvestre AM Miller C. The use of a prosthetic appliance to manage a large palatal defect in a dog. J Vet Dent. (1997) 14:61-4. 10.1177/089875649701400201 9571891

[33] Smith MM Rockhill AD. Prosthodontic appliance for repair of an oronasal fistula in a cat. J Am Vet Med Assoc. (1996) 208:1410-2. 8635989

[34] Thomas S. Leenstra AMK-JJCM. The healing process of palatal tissues after operations with and without denudation of bone: an experimental study in dogs. Scand J Plast Recons. (1999) 33:169-76. 10.1080/02844319950159415 10450573

[35] Leenstra TS Maltha JC Kuijpers-Jagtman AM Spauwen PH. Wound healing in beagle dogs after palatal repair without denudation of bone. Cleft Palate Craniofac J. (1995) 32:363-9. 10.1597/1545-1569_1995_032_0363_whibda_2.3.co_2 7578199

[36] Leenstra TS Kuijpers-Jagtman AM Maltha JC. The healing process of palatal tissues after palatal surgery with and without implantation of membranes: an experimental study in dogs. J Mater Sci Mater Med. (1998) 9:249-55. 10.1023/A:1008848509911 15348880

[37] Luskin IR. Reconstruction of oral defects using mucogingival pedicle flaps. Clin Tech Sm Anim Pract. (2000) 15:251-9. 10.1053/svms.2000.21044 11270002

[38] Nanci A. Oral mucosa. In: Nanci A editor. Ten Cate's Oral Histology. 9th ed. St. Louis: Elsevier (2018). p. 260-88.

[39] Lommer MJ Verstraete FJM Arzi B. Principles of oral oncologic surgery. In: Verstraete FJM Lommer MJ Arzi B editors. Oral and Maxillofacial Surgery in Dogs and Cats. 2nd ed. St. Louis: Elsevier (2020). p. 469-77.

[40] Folkman J. How is blood vessel growth regulated in normal and neoplastic tissue? Cancer Res. (1986) 46:467-73. 2416426

[41] Wallace J Matthiesen DT Patnaik AK. Hemimaxillectomy for the treatment of oral tumors in 69 dogs. Vet Surg. (1992) 21:337-41. 10.1111/j.1532-950X.1992.tb01707.x 1413465

[42] Emms SG Harvey CE. Preliminary results of maxillectomy in the dog and cat. J Small Anim Pract. (1986) 27:291-306. 10.1111/j.1748-5827.1986.tb02141.x

[43] Schusterman MA Harris SW Raymond AK Goepfert H. Immediate free flap mandibular reconstruction: significance of adequate surgical margins. Head Neck. (1993) 15:204-7. 10.1002/hed.2880150305 8387980

[44] Devalia HL Mansfield L. Radiotherapy and wound healing. Int Wound J. (2008) 5:40-4. 10.1111/j.1742-481X.2007.00351.x 18081782 PMC7951499

[45] Porock D Nikoletti S Cameron F. The relationship between factors that impair wound healing and the severity of acute radiation skin and mucosal toxicities in head and neck cancer. Cancer Nurs. (2004) 27:71-8. 10.1097/00002820-200401000-00009 15108954

[46] Peralta S Campbell RD Fiani N Kan-Rohrer KH Verstraete FJM. Outcomes of surgical repair of congenital palatal defects in dogs. J Am Vet Med Assoc. (2018) 253:1445-51. 10.2460/javma.253.11.1445 30451614

[47] Sinha UK Gallagher LA. Effects of steel scalpel, ultrasonic scalpel, CO_2_ laser, and monopolar and bipolar electrosurgery on wound healing in guinea pig oral mucosa. Laryngoscope. (2003) 113:228-36. 10.1097/00005537-200302000-00007 12567074

[48] Mison MB Steficek B Lavagnino M Teunissen BD. Comparison of the effects of the CO_2_ surgical laser and conventional surgical techniques on healing and wound tensile strength of skin flaps in the dog. Vet Surg. (2003) 32:153-60. 10.1111/j.1532-950X.2003.00153.x 12692760

[49] Davidson EB Davis MS Campbell GA Williamson KK Payton ME Healey TS. Evaluation of carbon dioxide laser and conventional incisional techniques for resection of soft palates in brachycephalic dogs. J Am Vet Med Assoc. (2001) 219:776-81. 10.2460/javma.2001.219.776 11561652

[50] Peralta S Manfra-Marretta S. Orofacial cleft repair. In: Verstraete FJM Lommer MJ Arzi B editors. Oral and Maxillofacial Surgery in Dogs and Cats. 2nd ed. St. Louis: Elsevier (2020). p. 392-403. 10.1016/B978-0-7020-7675-6.00049-8

[51] van de Wetering A. Repair of an oronasal fistula using a double flap technique. J Vet Dent. (2005) 22:243-5. 10.1177/089875640502200404 16494272

[52] Griffiths LG Sullivan M. Bilateral overlapping mucosal single-pedicle flaps for correction of soft palate defects. J Am Anim Hosp Assoc. (2001) 37:183-6. 10.5326/15473317-37-2-183 11300527

[53] Ellison GW Mulligan TW Fagan DA Tugend RK. A double repositioned flap technique for repair of recurrent oronasal fistula in dogs. J Am Anim Hosp Assoc. (1986) 22:803-8.

[54] Headrick JF McAnulty JF. Reconstruction of a bilateral hypoplastic soft palate in a cat. J Am Anim Hosp Assoc. (2004) 40:86-90. 10.5326/0400086 14736911

[55] Leon AC Davis LL Kraemer HC. The role and interpretation of pilot studies in clinical research. J Psychiatric Res. (2011) 45:626–9. 10.1016/j.psychires.2010.10.008 21035130 PMC3081994

